# Neurocognitive and Psychological Outcomes among Children and Adolescents with Brain Tumors: Development of an Observational and Longitudinal Prospective Study Protocol

**DOI:** 10.3390/bs13070536

**Published:** 2023-06-27

**Authors:** Giulia Zucchetti, Giorgia Gamberini, Sabrina Ciappina, Celeste Cagnazzo, Federica Ricci, Stefano Vallero, Paola Quarello, Paola Peretta, Franca Fagioli

**Affiliations:** 1 Pediatric Oncology and Hematology Division, Regina Margherita Children’s Hospital, 10126 Turin, Italy; giulia.zucchetti@unito.it (G.Z.); celeste.cagnazzo@unito.it (C.C.); stefanogabriele.vallero@unito.it (S.V.); paola.quarello@unito.it (P.Q.); franca.fagioli@unito.it (F.F.); 2 Section of Child and Adolescent Neuropsychiatry, Department of Public Health and Pediatric Sciences, Regina Margherita Children’s Hospital, University of Turin, 10126 Turin, Italy; giorgia.gamberini@unito.it (G.G.); federica.ricci@unito.it (F.R.); 3 Department of Public Health and Pediatric Sciences, University of Turin, 10126 Turin, Italy; 4 High-Intensity Surgery Division, Regina Margherita Children’s Hospital, 10126 Turin, Italy; paola.peretta@unito.it

**Keywords:** pediatric brain tumors, neuropsychological outcomes, central nervous system tumors, pediatric oncology, child development

## Abstract

Children and adolescents affected by brain tumors are at risk for neuropsychological sequelae that need to be evaluated in order to plan adequate rehabilitation programs, and to support their development and recovery. This work aims to describe an innovative prospective observational study protocol for the early evaluation and monitoring over time of neuropsychological outcomes in this pediatric population. Pediatric patients aged 3–17 with a brain tumor diagnosis will be assessed through the use of a battery of Italian standardized neuropsychological tests, with good psychometric properties and age-appropiate, at three different time points of their clinical course: at diagnosis and before surgery (T0), after surgical removal and before the start of potential adjuvant therapies (T1), and at the one-year follow-up after potential adjuvant therapies (T2). This study will allow clinicians to support the neuropsychological development of these children by promoting appropriate and timely rehabilitation and educational programs from the early phases of their clinical course.

## 1. Introduction

### 1.1. Background and Rationale

Pediatric brain tumors (BT) are the most common solid neoplasms in children, and the second most common malignancy of childhood after leukemia, representing approximately 25% of pediatric cancers and being a leading cause of cancer-related morbidity and mortality in this population [1]. While in adults most BT arises in the supratentorial region, about 60 to 70% of pediatric BT are infratentorial and develop in the posterior fossa. The most common types of BT in childhood are represented by astrocytomas, medulloblastomas, and ependymomas [2].

As survival among children treated for pediatric BT continues to improve, more attention is being focused on neuropsychological and long-term psychosocial outcomes related to BT and their treatments in order to improve their general development. Neuropsychological sequelae in this population may involve several functional domains and are related to different factors that may impact neurodevelopmental trajectories. These include the direct effect of BT and its localization, raised intracranial pressure, and treatments-related effects, from surgical intervention to adjuvant therapies (AT) such as radiotherapy and chemotherapy [3,4,5,6,7].

Functional recovery following acquired brain injury (ABI) is mediated by neuroplasticity, which includes both spontaneous reorganization and training-induced recovery [8]. The enhanced neuronal plasticity of the developing brain may involve both advantageous and detrimental effects on the recovery of children with ABI, such as for BT survivors [9]. Although a long-held belief, known as the “Kennard principle”, claims that recovery from brain damage will be more complete in the developing brain than in the adult brain, recent evidence suggests that this is not always true, as brain injuries have been found to be more severe if experienced in certain brain sites during critical periods of neural and cognitive development [10]. Recovery of function after brain injury in childhood depends upon both reparative and compensatory processes that are still poorly understood, and the more consolidated knowledge of adult neuroplasticity cannot necessarily be transferred to recovery after injury in the developing brain [11]. Indeed, unlike adults and adopting a Neuroconstructivist approach, BT in childhood could impact neuropsychological functioning both through impairment of specific areas of functioning at the time of diagnosis and treatments, and through the disruption of typical neurodevelopmental trajectories over the long-term period [12]. Thus, in children with BT, both the promotion of recovery of premorbid health and the support for the ongoing acquisition of developmental skills must be targeted in rehabilitation.

As regards the neurocognitive outcomes in these children, there is no unique neuropsychological phenotype that encompasses all tumor types, treatments, and other risk factors. However, attention, working memory and other executive functions, processing speed, learning, visuospatial and visuomotor functioning, and areas of academic achievement have been reported to be at particular risk [13]. It has been observed that BT are associated with less functional damage and with neuropsychological deficits that are more difficult to detect, compared to other more rapidly acquired brain injuries [14]. Moreover, neurocognitive impairments in this population often emerge slowly and increase over time, and it has been suggested that composite QI measures alone may not be sensitive to BT-related effects [15,16]. Thus, international guidelines strongly recommend including detailed neuropsychological assessments in research and clinical practice and monitoring these children over time from the diagnosis, through repeated follow-up evaluations [13,14,17]. This is fundamental in order to timely detect potential deficits, and to plan personalized rehabilitation and education programs aimed to reduce disability and improve child development.

In the last decades, research evidence of neurocognitive sequelae in this population has increased [5,6,7,8,9,10,11,12,13,14,15,16,17,18]. However, most research studies are not longitudinal and typically include heterogeneous populations with respect to the types of treatments and diagnosis-assessment times [18,19,20]. Moreover, few studies to date have described neuropsychological outcomes before surgical resection in the early postoperative phase before AT [21,22].

### 1.2. Objectives

The primary aim of this study protocol is to assess neuropsychological outcomes in pediatric BT patients at different time points of the clinical course: at diagnosis and before surgery (T0), after surgical removal and before the start of potential AT (T1), and at the one-year follow-up after potential AT (T2). Possible changes over time in neuropsychological functioning in our cohort will be considered through the comparison of neuropsychological data of the three longitudinal assessments. Moreover, our study aims to evaluate the impact of different relevant factors on neuropsychological outcomes in this population, such as tumor location and type of treatment. The role of different demographic and clinical variables will be analyzed, and potential relevant prognostic factors related to worse neuropsychological outcomes will be identified.

### 1.3. Study Design

This study protocol is designed as a prospective, cohort observational study with a longitudinal design. Children and adolescents with a newly diagnosed BT will be monitored over time, and the neuropsychological outcomes in this cohort will be assessed at three different time points: (a) in the preoperative phase, (b) in the early postoperative phase before the start of any ATs, if required, and (c) at follow-up one year after the postoperative assessment and following ATs, if required. The study will be conducted and reported according to STROBE guidelines [23]. The present study protocol is described following the SPIRIT guidelines and checklist [24].

## 2. Methods

### 2.1. Study Setting

The participants considered for the study will be children and adolescents admitted at the Pediatric Oncology and Hematology Division and High-Intensity Surgery Department of the Regina Margherita Children’s Hospital in Turin, Italy. Before admission and after discharge from intensive care units, neuropsychological evaluations will be carried out. Assessments will be performed by a trained clinical psychologist and a pediatric neuropsychologist in a one-to-one setting with the child.

Our academic hospital is one of the main centers of the Italian Association of Pediatric Hematology and Oncology (AIEOP). AIEOP is an Italian Scientific Society aimed at providing equal structural, medical, and assistance resources for pediatric patients diagnosed with tumors. The AIEOP was founded 40 years ago and now consists of 49 pediatric oncology centers around Italy, dedicated to clinical care and research in the field of pediatric cancer. However, there is still a lack of homogeneity in the quality of care throughout different Italian AIEOP centers. Indeed, optimal care is only provided by a few highly specialized hospitals in certain regions, and this causes a certain degree of patient migration towards these centers, which means patients and their families have to leave their hometowns in order to receive the best care. Therefore, to cope with patient migration and to be aligned with European standards, some Pediatric Oncology Regional Networks are trying to adopt the hub-and-spoke model in order to achieve greater uniformity in the provision of care. Piedmont was the first region in Italy to adopt the hub-and-spoke network model in which the Regina Margherita Children’s Hospital in Turin is the hub center. As the reference center of the network, our hospital works with nine spokes centers located throughout Piedmont. The spokes consist of three second-level units and six first-level units, which only offer essential facilities. In this hub-and-spoke network model, patients with BT aged zero to eighteen years are followed from diagnosis to the survivorship phase or end-of-life. Lastly, the Surgery Division of our hospital represents a reference center in our region and is the only one to be specialized in pediatric neurosurgery in this area. New technological advancements have improved the safety and outcomes of neurosurgical procedures, with the goal of a safe and maximal resection while minimizing neurological deficits and possible neuropsychological sequelae. After neurosurgery, children who require AT, including CT, RT, or a combination of both, are admitted to our Oncology department, where these treatments are delivered. The non-surgical management of these patients is complex and depends upon several factors, such as the age of the patient, histologic diagnosis, degree of residual tumor, presence or absence of dissemination, and availability of clinical trials in our center.

### 2.2. Participants and Eligibility Criteria

Children and adolescents with a diagnosis of BT will be recruited to participate in the study. Based on the epidemiology of BT among children and adolescents in the study site, a minimum of fifty patients will be enrolled in the overall protocol every three years, according to the following inclusion and exclusion criteria. To be eligible for the study, participants must be aged between three and seventeen years old at the time of diagnosis. Children will be excluded if they meet the following exclusion criteria: (1) presence of BT recurrence or secondary surgery/treatment after completion of surgical removal and/or AT in the past; (2) presence of a certified premorbid neurological or neurodevelopmental disorder; (3) presence and persistence of severe motor/neurologic/sensory deficits that enable to complete a formal neuropsychological assessment; (4) inadequate knowledge of Italian language; (5) lack of consent.

As we are interested in describing neuropsychological outcome in children with BT in the preoperative and early postoperative phase before the start of any AT, children with BT recurrence that already had surgical removal, and/or AT in the past will not be included because of the known long-term effects of these treatments on neuropsychological functioning [5]. Children with other premorbid neurological and developmental disorders will not be considered as these may be associated with premorbid neuropsychological sequelae that would add to those related to BT and its treatment. Patients with persisting and severe neurologic/motor/sensory sequelae will be excluded as these could interfere with the child’s ability to sustain the formal assessment.

Eligibility will be assessed through the screening of electronic medical records and clinical-anamnestic interviews with caregivers.

### 2.3. Participant Timeline

**Figure 1:**
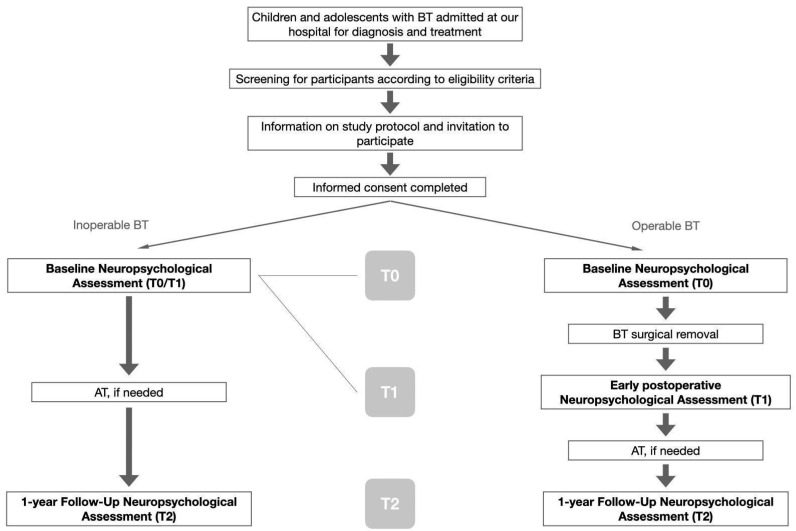
Study protocol flowchart.

Participants will be monitored longitudinally over time and will complete neuropsychological assessments at three different time points (Figure 1):

(a) Preoperative assessment (T0): after diagnosis and before BT surgical removal. This evaluation is aimed to identify possible tumor-related effects on neuropsychological functioning, excluding the outcomes related to surgical removal or AT.
(b) Postoperative assessment (T1): in the early phase after surgery and before the start of potential adjuvant therapies. This assessment is intended to describe tumor and surgery-related neuropsychological outcomes in our cohort. Early evaluations also allow for planning prompt and adequate rehabilitation programs.
(c) One-year follow-up assessment (T2): one year after T1 and the beginning of potential AT, if necessary. This assessment is aimed to describe the long-term neuropsychological outcomes that may also include initial AT-related effects and potential neurocognitive decline.

In case of inoperable BT, the participant will complete only one neuropsychological assessment after diagnosis and before the start of potential AT (T0 = T1).

### 2.4. Outcomes

International guidelines for the identification and interventions for neurocognitive sequelae in children with BT suggest evaluating all the neuropsychological domains that typically are mainly affected in this clinical population. While BT survivors may present impairments in any area of functioning depending on lesion site and secondary neurological complications, there are nonetheless specific functions that are more commonly impaired due to white matter changes and neurotoxicity, such as global cognitive functioning, attention and executive functions, processing speed, memory and visuo-perceptual skills. They also highlight the importance of providing a baseline assessment to all pediatric BT patients at the time of diagnosis and in the early post-operative phase, even in the absence of any overt manifestation of CNS injury, in order to better guide rehabilitation and understand the neuropsychological outcomes and possible cognitive decline that may be observed over time at long-term follow-ups [13]. Moreover, they suggest incorporating in standard clinical care repeated neuropsychological assessments over time through brief and tailored evaluations. Ideally, they propose to develop a model that includes a brief but comprehensive screening battery with psychometrically robust tests, to be followed, when necessary, according to clinical judgment, by a full neuropsychological battery aimed to deepen and better understand the observed impairments [13,14].

In accordance with international guidelines [13,14], the neuropsychological battery is aimed to evaluate different areas of functioning and includes tools for the assessment of the impact of the tumor and its treatment on affective/behavioral, adaptive functioning and quality of life. Outcome measures include different Italian standardized neuropsychological tests, with good psychometric properties, internationally used and age appropriate. The battery aims to be comprehensive, but also of relatively brief administration and feasible in order to reduce participants’ and families’ burden [9]. The core battery consists of both direct and indirect tools and is intended to rapidly screen and identify possible neuropsychological deficits. Possible difficulties will then be investigated through additional and optional tests according to clinical judgment. Fundamental domains for assessment have been identified based on existing evidence of main neurocognitive sequelae in children surviving BT with potential risk for neurotoxicities [13,14,15,18]. Besides intellectual and overall cognitive functioning, basic neuropsychological processes such as attention and executive functions will be considered, as well as different higher-order functions such as language, learning and memory, visuospatial abilities and social cognition. Social cognition (SC) outcomes, that encompass, for example, emotion recognition and theory of mind abilities, have been included in this study because of their clinical relevance for the child’s socio-relational development and because, to date, evidence on SC competence in pediatric BT population is still very limited [25,26]. In the last decades, most of the studies on neurocognitive sequelae in pediatric BT population have typically used Wechsler intelligence scales and IQs. However, recent studies have recently emphasized the importance of more comprehensive assessments, as intelligence scales alone do not seem sensitive enough for detecting neuropsychological deficits in pediatric-acquired brain injury and BT patients, thus proving inadequate for planning personalized rehabilitation programs and supporting school reintegration [27]. 

In the Preoperative Assessment (T0), the battery consists of different indirect questionnaires for caregivers. In this delicate clinical phase, a direct formal assessment of the child is often challenging because of the neurological symptomatology and treatment urgency. In the following assessments at the postoperative phase (T1) and at the one-year follow-up (T2), the same questionnaires for caregivers will be proposed together with the core battery of direct tests. A detailed description of the outcome measures and of their use in the different time points is outlined in the following table (Table 1). All outcome measures collected will be converted into standard scores (Z-scores, scaled-scores, or T scores) according to each test scoring procedure and based on age-corrected normative data.

### 2.5. Recruitment

Participants will be recruited from the Pediatric Oncology and Hematology Division and High-Intensity Surgery Department of Regina Margherita Children’s Hospital in Turin, Italy. After an initial screening for general exclusion criteria, eligible participants and their caregivers will be directly contacted by members of the research group during admission and will receive information on the study protocol. A second and more detailed screening of potential participants will be made on relevant clinical information collected during clinical interviews with caregivers and child observation. Written informed consent will be obtained by caregivers for agreement to participate in the study, and all children will provide their willingness to take part in the study. Recruitment started in November 2022 and will finish in October 2025.

### 2.6. Data Collection

Data collection will be carried out at the Child and Adolescent Neuropsychiatry Department and Psycho-Oncology Unit of the hospital. Assessments will be performed by a pediatric neuropsychologist and a trained clinical psychologist, after a training period aimed at ensuring homogeneity in administration and scoring procedures between the two assessors. Assessment is estimated to take about three to four hours to complete, for a total of three to four one-hour sessions in a one-to-one setting with the child. The requests will be adapted to the needs and clinical condition of each patient, and the evaluation will be carried out in different sessions in order to reduce the child’s fatigue and attentional drops. Demographic and clinical data will be collected through electronic medical records and clinical-anamnestic interviews with caregivers. Checklists will be used to monitor data collection and all patient data will be recorded on a Case Report Form. The following demographic and clinical data will be collected for each participant: age at diagnosis, sex, parental SES, education, handedness, bilingualism, premorbid functioning, tumor localization and histology, tumor grade and size, presence of hydrocephalus and shunt procedures, preoperative intracranial hypertension, neurosurgical technique, MRI/CT data, neurological symptoms, secondary neurological complications, severity and duration of Cerebellar Mutism Syndrome [43] for the infratentorial subgroup, type and duration of RT/CT, pharmacological therapy, presence of aphasia, sensorial and/or motor disturbances.

### 2.7. Data Management

Study data will be stored as a combination of paper and electronic files and then entered into a hospital database, which acts as an electronic CRF. Data will be held, administered, and analyzed according to study standard operating procedures. Collected data will be securely stored and identified by ID number only. All confidential participant contact information and identifiable data will be stored separately within the database. All study documents will be stored in accordance with relevant government regulations regarding the retention and disposal of participant records.

### 2.8. Statistical Analysis Plan

Statistical analysis will be performed on average standard scores at group level for each outcome measure. Demographic and clinical variables of the sample will be described through descriptive statistics (percentages, means, medians). To evaluate the presence of neuropsychological deficits, differences between our sample and normative data will be analyzed by independent sample *t* tests (two-tailed) for continuous variables and by χ^2^ for categorical variables for each time point (T0, T1, T2). The Shapiro-Wilk test will be used to check the normal distribution of data. Comparisons in neuropsychological functioning and change over time (T0 vs. T1 vs. T2) at group-level will be analyzed through the use of repeated measures of ANOVA. When an adequate sample size has been reached, the ANCOVA analysis will be used in order to investigate the possible effects of different relevant variables (age at diagnosis, SES, BT grade and size, baseline IQ) on neuropsychological outcomes at the different time points. Subgroup analyses will be in order to identify possible other effects such as treatment strategies (surgery-only vs. RT/CT). Corrections for multiple comparisons will be performed through the use of the Hochberg and Hommel methods, or the step-down miniP method [44].

### 2.9. Ethics and Dissemination

The study has been approved by the Ethical Committee of our hospital in October 2022 (Protocol Number: 0001417). The study will be conducted according to Good Clinical Practice guidelines [45] and Helsinki Declaration. Caregivers will be provided with detailed information on study participation and will complete written informed consent. For children and adolescents between the age of six- and eighteen-years, consent to participate will be obtained. Study participation is completely voluntary and patients that will not participate will receive all the standard clinical care and treatments provided by our hospital. Participants and caregivers will be informed of the study results at the end of the study. After each neuropsychological assessment, evaluation results will be explained to caregivers and participants during a clinical interview and a written neuropsychological report will be delivered. Neuropsychological outcomes will inform and guide the rehabilitation recommendations for each participant. Research findings will be published in peer-reviewed journals and will form part of a PhD thesis. All study data will be de-identified and analyzed at a group-level in order to protect the privacy of participants and ensure confidentiality. Relevant anonymized participant data will be shared with the research team for research and clinical purposes related to the study.

### 2.10. Expected Results and Clinical Implications

Through this study protocol, we expect to better characterize the neuropsychological and emotional-behavioral sequelae that may be associated with pediatric BT, with a particular focus on the early stages of their clinical course. Indeed, research evidence on early neuropsychological impairments that emerge in the pre-operative phase and the early post-operative phase is still very limited to date, and most observational studies in this field focused on long-term outcomes [18]. It can be expected that neuropsychological deficits may be detected since these early phases, that in this case would be related to the effects of a growing mass on the central nervous system and to the effects of the surgical removal of the tumor. We hypothesize that neuropsychological outcomes will be worse when potential risk factors are present, such as younger age, secondary neurological complications, higher tumor size and grade, presence of post-operative complications such as Cerebellar Mutism Syndrome [43] for infratentorial tumors, and completion of AT. From a longitudinal perspective, it is possible to hypothesize that, in the low-grade tumor subgroup that typically receives surgery without subsequent AT, neuropsychological outcomes may remain unchanged or even improve over time at the one-year follow-up assessment compared to the early post-operative one, especially when rehabilitation programs are activated in between. On the contrary, with respect to the high-grade tumor subgroup that typically receives RT and/or CT, we may expect that initial signs of progressive neurocognitive decline may emerge at the one-year follow-up evaluation [5]. Lastly, considering the infratentorial subgroup, we expect that neuropsychological and affective-behavioral sequelae that may emerge may be in line with cognitive and affective deficits due to cerebellar damage and cerebo-cerebellar tracts involvement described by the Cerebellar Cognitive Affective Syndrome [46]. Impairments observed in this subgroup may be worse if associated with higher severity and longer duration of post-operative Cerebellar Mutism Syndrome [43].

Beyond contributing to increasing research evidence in the field of neurocognitive outcomes of pediatric BT, this study will allow clinicians to support the neuropsychological development and functional recovery of these children through the planning of timely and personalized rehabilitation and educational programs, from the early phases of clinical course. Most of the centers that deal with the clinical care of children with BT adopt a multidisciplinary approach that addresses the neurological, motor and linguistic outcomes through professionals such as oncologists, child neuropsychiatrists, and speech and motor therapists. Moreover, as the psychological distress associated with this kind of diagnosis is considerable, psychological support for children and their families is typically provided over the clinical course. Despite the importance of neuropsychological monitoring in pediatric neuro-oncology has been recommended and stressed by international guidelines [13], the figure of neuropsychologists is still lacking in most of the pediatric centers, and neuropsychological outcomes in this clinical population are still overlooked [14,17]. This study protocol has the purpose of filling this clinical gap, allowing adequate monitoring of neuropsychological sequelae that these children may manifest since the early post-operative phase and informing rehabilitation professionals about timely and personalized rehabilitation programs. Early interventions have the potential both to promote functional recovery and improve impaired functions and to prevent progressive neurocognitive decline associated with AT. To this purpose, a constant collaboration among psychologists, neuropsychologists, caregivers, rehabilitation facilities and schools will be maintained from diagnosis to long-term follow-ups. A constant dialogue between clinicians and schoolteachers is fundamental for a positive reintegration at school; information about possible consequences of BT in general and about the neuropsychological functioning of each child should be provided to teachers, with the purpose of finding personalized strategies to support their learning abilities and academic skills. In general, this protocol may contribute to improving the long-term outcomes of children and adolescents with BT that will take part in the study, with the goal of ameliorating their quality of life.

In conclusion, this study protocol on neuropsychological monitoring will contribute both to increasing the research evidence in the field of neuropsychological outcomes in pediatric neuro-oncology and to improving the quality of the clinical care provided to pediatric patients with BT. Lastly, observational studies aimed to better characterize the neuropsychological sequelae that children with BT may manifest are fundamental for future research, as they provide useful information that may guide the development of new neuropsychological rehabilitation programs specifically dedicated to this clinical population, whose effectiveness may be evaluated through future experimental studies.

## Figures and Tables

**Table 1:** Neuropsychological assessment battery. List of standardized tests with references: WISC-IV, WPPSI-IV, WAIS-IV [28,29,30,31]; NEPSY-II [32,33]; BVN 5-11, BVN 12-18 [34,35]; BAFE [36]; BRIEF-P, BRIEF-2 [37,38,39]; ABAS-II [40]; CBCL [41]; PedsQL [42].

Full Neuropsychological Assessment Battery					Time Points		
Domain	Function	Measure	Administration	Age Range	T0	T1	T2
Intellectual functioning	Global cognitive functioning	Wechsler Scales (WPPSI-IV, WISC-IV, WAIS-IV)	Child	3–18		X	X
Attention	Visual selective and sustained attention	Visual Attention-NEPSY-II	Child	3–16		X	X
		Visual Attention-BVN 12–18	Child	17–18		X	X
	Auditory selective and sustained attention	Auditory Attention and Response Set-NEPSY-II	Child	3–16		X	X
		Auditory Attention-BVN 12–18	Child	17–18		X	X
Executive Functions	Verbal inhibition	Day&Night-BAFE	Child	3–5		X	X
		Inhibition-NEPSY-II	Child	6–16		X	X
	Verbal working memory	Digit span backward-Wechsler scales (WISC-IV, WAIS-IV)	Child	6–18		X	X
		Digit span backward-BVN 5–11	Child	5		X	X
	Visual working memory	WM Index-WPPSI-IV	Child	3–5		X	X
	EF ecological assessment	BRIEF-P	Parent report	3–5	X	X	X
		BRIEF-2	Parent report	6–18	X	X	X
Memory	Verbal short-term memory	Digit span forward-Wechsler scales	Child	6–18		X	X
		Digit span forward-BVN 5–11	Child	5		X	X
	Verbal long-term memory and learning	Word List-BVN 5–11	Child	5–6		X	X
		List Memory-NEPSY-II	Child	7–16		X	X
		Word List-BVN 12–18	Child	17–18		X	X
Language	Verbal Fluency	Word Generation-NEPSY-II	Child	3–16		X	X
		Verbal Fluency-BVN 12–18	Child	17–18		X	X
	Verbal comprehension	Comprehension of Instructions-NEPSY-II	Child	3–16		X	X
Visuospatial abilities	Visuoperceptual abilities, mental rotation	Geometric Puzzles-NEPSY-II	Child	3–16		X	X
	Constructional praxis	Block design-Weschler scales	Child	3–18		X	X
Social Cognition	Emotion recognition	Emotion Recognition-NEPSY-II	Child	3–16		X	X
	Theory of mind abilities	Theory of Mind-NEPSY-II	Child	5–16		X	X
Adaptive functioning and daily living		ABAS-II	Parent report	3–18	X	X	X
Behavioral/emotional functioning		CBCL 1.5–5, CBCL 6–18	Parent report	3–18	X	X	X
Quality of life		PedsQL-Core	Parent report	3–18	X	X	X

## Data Availability

Data available on request due to ethical and privacy restrictions. The data presented in this study will be available on request from the corresponding author. The data will not be publicly available due to privacy restrictions.
